# Spontaneous Yawning and its Potential Functions in South American Sea Lions (*Otaria flavescens)*

**DOI:** 10.1038/s41598-019-53613-4

**Published:** 2019-11-21

**Authors:** Elisabetta Palagi, Federico Guillén-Salazar, Clara Llamazares-Martín

**Affiliations:** 1 Unit of Ethology, Department of Biology, University of Pisa, Via A. Volta 6, 560126 Pisa, Italy; 2 Natural History Museum, University of Pisa, Via Roma 79, 56011 Calci, Pisa Italy; 3 Ethology and Animal Welfare Section, Universidad Cardenal Herrera CEU, CEU Universities, c/Tirant lo Blanc 7, E-46115 Alfara del Patriarca, Valencia Spain

**Keywords:** Animal behaviour, Animal physiology

## Abstract

Spontaneous yawning is a widespread behaviour in vertebrates. However, data on marine mammals are scarce. In this study, we tested some hypotheses on the functions of yawning in a captive group of South American sea lions (*Otaria flavescens*). According to the *Dimorphism Hypothesis*, species showing low levels of sexual dimorphism in canine size do not show sex differences in yawning distribution; this was supported by our findings, since yawning did not differ between the sexes. Yawning was more frequently performed during resting/sleeping contexts, thus supporting the *Drowsiness Hypothesis*. Yawning and self-scratching are considered reliable indicators of short-term anxiety in sea lions, since they immediately increased after conflicts both in aggressors and victims (*Social Distress Hypothesis* supported). In the long-term, yawning was not correlated with individuals’ dominance status, thus showing that anxiety is similarly experienced by dominants and subordinates. The last two findings can be explained by the social competition of this species, that involves individuals independently from their sex, age or ranking status. Therefore, the exposure to frequent stressful events can induce similar levels of anxiety in all the subjects (*Resource Inequity Hypothesis* supported). In conclusion, spontaneous yawning in sea lions seems to share similar functions with other social mammals, suggesting that this behaviour is a possible plesiomorphic trait.

## Introduction

Yawning is a ubiquitous behaviour present in all classes of vertebrates^1^. It is a stereotyped behaviour that once triggered, is uncontainable and unstoppable^2^. In air-breathing animal species, the first phase of a yawning event is characterized by a slow and wide opening of the animal’s mouth and is accompanied by a deep inhalation. The second phase includes a quick closure of the mouth and a short exhalation^3^. Depending on the species, yawning may be also accompanied by eye closing, vocalizations, body stretching, pandiculation and even tongue protrusion^4–7^.

Many hypotheses regarding the potential functions of spontaneous yawning have been proposed. These hypotheses can be classified into two groups, the physiological and social hypotheses. In the former group, yawning is posited to act as a homeostatic restoring mechanism, and, with the intensification of the studies, some of these hypotheses such as brain cooling, anxiety and drowsiness hypothesis have found an increasing support^3,4,8–14^. In the latter group, the performance of yawning is posited to express the emotional state of the yawner as a communicative tool (*e*.*g*., threat yawns), since other members of the group would be able to associate it with certain contexts or behaviours and so could act accordingly^12–15^. Moreover, yawning has been proven to have an infectious nature (contagious yawning), suggesting that it may be a primitive form of emotional contagion^5,16,17^.

Spontaneous yawning is mainly displayed during resting contexts characterized by the absence of changes in social and environmental stimuli^2,6^. The effect of yawning can be linked to a state of drowsiness in which animals may change from an awake to a sleep phase and vice versa^1^. It has been proposed that the role of yawning in such situations is to increase the alertness state, thus making animals able to respond effectively to sudden and urgent situations^18^.

Spontaneous yawning can also be affected by social stimuli and conditions^14,15,19,20^. For example, in birds^4,21^, rats^22^, lemurs^13^ and monkeys^12,20,23^, yawning increases after an anxiogenic event which can affect the homeostasis of the subject. In the wild, Goodall^24^ observed that chimpanzees yawned more frequently in the presence of human observers and, in captivity, the presence of humans in front of animal facilities produced an increase of yawning in monkeys (lion-tailed macaques, *Macaca silenus*^23^). For this reason, along with self-directed behaviours such as self-grooming and self-scratching, yawning can be considered an indicator of anxiety^25,26^. For example, in chimpanzees, Baker and Aureli^27^ showed that individuals tended to yawn and self-scratch more frequently during high social tension conditions that provoked an increase of anxiety and arousal in the subjects. Recent findings suggest a possible effect of yawning as a stress-releaser, which helps restoring physiological/emotional homeostasis^4,28,29^.

In species showing an evident sexual dimorphism in their canine size, yawning is more frequent in males than in females^30^. The development of canines in males is a secondary sexual characteristic that provides them advantages both in intra- and inter-sexual competition^31^. The canine exposure during a yawning event may convey information about the transitional phase experienced by the yawner that can pass from a relaxed to a tense emotional state or vice versa. Hence, yawning can anticipate a forthcoming aggressive behaviour (“threat displays”^20,32,33^) or a reduction of the levels of anxiety in the yawner.

In-group conflicts are undoubtedly a source of social tension for both aggressors and victims, which often increase self-directed behaviours in the immediate period after an agonistic event^13,25,26^. This is also true for yawning, which shows a peak in the minutes after the end of an agonistic contact (Nazca booby, *Sula granti*^21^; ring-tailed lemur, *Lemur catta* and Verreaux’s sifaka, *Propithecus verreauxi*^13^; macaques, *Macaca tonkeana*^20^).

This wide array of results strongly suggests the multifunctional nature of this behaviour, which has been widely studied in primates and rodents, but has often been neglected in other species such as marine mammals. Here, we test some hypotheses, not necessarily mutually exclusive, on spontaneous yawning in South American sea lions, a species living in harems and characterized by high levels of competition^34–38^. Sea lions show a strong sexual dimorphism in body size and morphology (males possess a mane around their heads), but show a reduced sexual dimorphism in canine length. The *Sexual Dimorphism Hypothesis* predicts that yawning is more frequent in males when the species presents evident sexual differences in canine size^12,20,27^. We thus predict that males and females of South American sea lions do not necessarily differ in the performance of yawning (Prediction 1).

In species showing an unequal distribution of social power, subordinates experience greater levels of anxiety in the long-term^39^. Within social groups, hierarchy affects stress levels, which can be evident from the baseline frequency of self-directed behaviours (*e*.*g*., with the dominants scratching more than subordinates or vice versa). The self-scratching trend across hierarchy depends on social organization in different species or populations, with subordinates showing the highest long-term stress levels in hierarchically stable social groups of despotic species^39^. According to the *Resource Inequity Hypothesis*^39^, since yawning is a reliable indicator of stress, it will be more frequently displayed by subordinate than by dominant individuals, reflecting a baseline anxiety in these kinds of social groups^20,26,39^. For example, in species such as Japanese macaques (*Macaca fuscata*) that are characterized by strong power asymmetry, yawning reflects a baseline anxiety state in subordinate individuals that show higher yawning frequencies than in dominant individuals^20^, particularly during periods of social stability. Although South American sea lions establish dominance hierarchies between group members, a strong competition over resources is present at all ages and in both sexes (*e*.*g*., females, shade areas for nursing or water access for thermoregulation), thus reducing the monopolization of resources by high ranking individuals^34–38,40^. For this reason, we expect that yawning does not strictly follow a dominance gradient in this species (Prediction 2).

If yawning and self-scratching are behavioural responses linked to an anxiety state of South American sea lions in response of an immediate perturbing event^4,22^ (the *Social Distress Hypothesis*), we expect that after an agonistic event, these two behavioural patterns will increase by following a similar trend in both the aggressor and the victim (Prediction 3).

Finally, if yawning, as it occurs in other mammal species^12,13^, is a mechanism linked to the alertness state of subjects (the *Drowsiness Hypothesis*), we expect it to be concentrated during periods of inactivity characterized by an alternation of resting and sleeping phases (Prediction 4).

## Methods

### Subjects and housing

We collected behavioural data on a group of South American sea lions (*O*. *flavescens*) housed at the *Oceanogràfic* aquarium (Valencia, Spain). The group was made up of one harem of 18 individuals, but only the behaviour of 13 individuals was taken into consideration (1 adult male, 8 adult females, 2 juvenile males and 2 juvenile females) since infants were excluded from the analysis (Table 1). Kinship between subjects was known. The animals were held in an outside enclosure formed by three rocky platforms (one large peninsula and two small islands) surrounded by a pool (1078 m^3^). Sea lions were free to move from one place of the enclosure to another throughout the day and night. They were fed three times a day (at 10.15 a.m., 1 p.m. and 4.30 p.m.). Fish were scattered in the water by the keepers in order to make the feeding more challenging and as natural as possible for sea lions. There were no blind spots in the enclosure. Animals were trained only for medical purposes and did not take part in any show. Abnormal behaviours were never observed during the study.

**Table 1:** Composition of the South American sea lion group housed at the *Oceanogràfic* aquarium during the period of data collection.

Subjects	Code	Sex	Age	NDS_values_	Mean (Nº of Ys/h)	Y duration (seconds) (mean ± SE)
Alvin	1	♂	13 yr	9.46	3.53	5.43 ± 0.24
Patrick	2	♂	4 yr	8.00	3.49	3.72 ± 0.15
Demmy	3	♀	13 yr	5.62	1.70	4.31 ± 0.47
Laura	4	♀	13 yr	6.13	2.51	5.31 ± 0.33
Selkie	5	♀	13 yr	5.99	3.00	4.40 ± 0.20
Ambar	6	♀	13 yr	5.76	3.14	4.66 ± 0.22
Morena	7	♀	13 yr	6.10	3.62	5.64 ± 0.46
Janis	8	♀	4 yr	5.83	2.11	3.61 ± 0.40
Portos	9	♂	3 yr	3.76	2.43	3.24 ± 0.23
Nora	10	♀	3 yr	3.99	2.89	4.00 ± 0.18
Ana	11	♀	13 yr	4.87	2.77	4.68 ± 0.29
Naima	12	♀	13 yr	5.36	4.00	3.83 ± 0.14
Noa	13	♀	13 yr	7.14	2.10	4.77 ± 0.53

NDS = Normalized David’s Scores; yr = years; Y = Yawn.

### Data collection

Before the systematic data collection, the observer (C.LL-M.) underwent a training period so as to be able to identify the animals of the colony. Subjects were identified by their external features (fur colour, scars and facial physiognomy) as well as by their individual characteristics (sex and age).

The observational period lasted 14 months (May, 2013 - July, 2014). A total of 304 continuous focal samplings^41^ were collected during this period. Each sampling lasted 6 minutes and individuals were, as much as possible, recorded in balanced proportions. Focal samplings were recorded using a video camera (Sony DCR-SR21), with 29 h of video collected. Videos were recorded between 3 p.m. and 9.30 p.m., as that was the time interval in which animals spent more time out the water. The video framing covered a maximum of 4 metres around the focal subject in order to collect social interactions with good video definition.

### Operational definitions and video analyses

**Figure 1:**
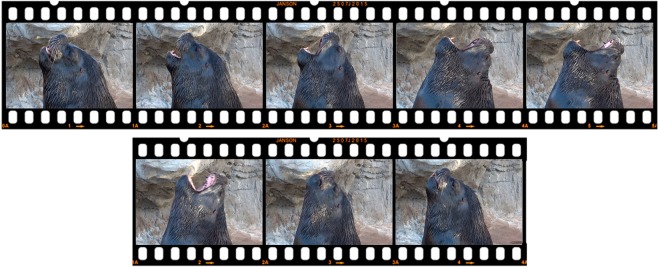
Yawning sequence. At the top, the long inspiration phase and at the bottom, the exhalation phase of a shorter duration. (Photos by C. Llamazares-Martín).

Videos were analyzed with VLC 2.1.5 Rincewind software with an accuracy of 0.4 seconds and Kinovea-0.7.10 software. We used the all-occurrences sampling rule^41^ in order to obtain all yawning events from the videos. We defined a yawn as when a sea lion opened its mouth, while simultaneously inhaling deeply, until its mouth opened to its maximum. At this time, its vibrissae were erect, its lips retracted, and its teeth were partially exposed. Mouth closing and air exhalation were faster than mouth opening and inhalation. Yawns were sometimes followed by swallowing and by a slight protrusion of the tongue (Fig. 1).

When a yawn occurred, we registered the identity of the yawner, the time and duration of the yawning event, and the yawner’s posture (defined as lying, sitting or walking).

Yawns were classified as occurring in four types of contexts: when resting/sleeping, and when engaged in affiliative, play-fighting or agonistic behaviour. During the resting/sleeping context, the animal was not involved in any social interaction, and remained lying down, shifting from an awake to a sleeping phase. If the subject was involved in a social interaction with conspecifics, and was engaging in rubbing, body contact or nose-nose contact we classified that context as affiliative. When animals were engaged in social play, the context was labelled as play-fighting. Finally, when the subjects were involved in or witnessed an aggressive interaction, such a context was categorized as agonistic^34,35^ (starting from the beginning of the aggression to one minute after the end of the aggressive event).

Inter-observer reliability in characterizing and scoring behaviours patterns was tested by E.P., who randomly selected parts of the dataset and checked whether the behavioural categories/patterns were correctly classified from the videos. Such checking was carried out for each month of data collection (14 times in 8 months of video analysis). The Cohen’s kappa values for the behavioural categories were 0.84 for agonistic, 0.88 for affiliative, 0.85 for play-fighting and 0.86 for resting/sleeping. As for the behavioural patterns, the Cohen’s kappa values were 0.89 for yawning and 0.90 for self-scratching.

### Hierarchy analysis

We calculated the steepness of the dominance hierarchy from matrices of decided conflicts via Steepness 2.2^42^. Normalized David’s scores (NDS) were calculated according to a dyadic dominance index (Dij) in which the registered proportion of wins (Pij) is corrected for the chance occurrence of the observed outcome, which is calculated on the basis of a binomial distribution with all animals having same chances of winning or losing dominance encounters^43^. The correction was applied since there was a strong variability in the frequency of interactions between dyads. The NDS-based hierarchy was created by ordering the subjects according to their NDS.

### Analysis of the yawning distribution according to age, sex, NDS and context

We ran a Linear Mixed Model (LMM) to determine which variables affected yawning distribution. The dependent variables were the hourly frequency of yawning and its duration (Normal distribution, Anderson-Darling, ns, EasyFit 5.5 Professional). The identity of the subjects (nominal variable) was included as the random factor. The fixed factors were age (adults, juveniles), sex (males, females), NDS values and contexts (resting/sleeping, affiliative, play-fighting and agonistic).

We tested all possible combinations of the fixed variables considered, spanning a single-factor model and a model including all the fixed factors (full model). To select the best model, we used the Akaike’s corrected information criterion (AICc, for small sample sizes). We calculated the difference (ΔAICc) between the AIC_C_ value of the best model and the AIC_C_ value for each of the other models. As suggested by Symonds and Moussalli^44^ (p. 17) ‘… as a coarse guide, models with ΔAICc values less than 2 are considered to be essentially as good as the best model, and models with ΔAICc up to 6 should probably not be discounted. Above this, model rejection might be considered, and certainly models with ΔAICc values greater than 10 are sufficiently poorer than the best AIC model as to be considered implausible’. Moreover, to assess the relative strength of each model, we employed ΔAICc to calculate the evidence ratio and the Akaike weight (*w*i). The *w*i (ranging from 0 to 1) is the weight of evidence or probability that a given model is the best model, taking into account the data and set of candidate models^44^.

### Post-conflict/Matched-control observations (PC/MC)

To verify if yawning and self-scratching can express a distress emotional state in the performer^26^ (*e*.*g*., anxiety), we applied the post-conflict and matched-control method (PC/MC)^45^. For post-conflict (PC) data collection, after the last occurrence of an aggressive pattern for a given agonistic encounter, the behaviour of the victim and the aggressor was recorded for 30 seconds. Each PC observation was followed by a 30-sec matched-control (MC) observation, which was made in another video in which the same animals were present (aggressor and victim), in the absence of agonistic interactions during the 30 seconds before the beginning of the observation and in a similar social context. During PC and MC observations, we recorded each yawning and self-scratching event performed by the aggressor and/or the victim. Self-scratching occurs when an animal uses its claws or teeth to rub its fur repetitively and contact its skin. We recorded a total of 159 PC/MC observations for the aggressor and 91 PC/MC observations for the victim. The difference in the number of PC/MCs for the aggressor and the victim is due to the fact that, after an aggressive event, the victim often fled, jumping into the water, thus preventing it from being observed the following 30 seconds. Due to the non-normality of the data (KS < 0.05), we compared the two conditions PC vs MC via the Exact Wilcoxon Signed Rank test. All data were analysed at the individual level and were two-tailed. The significance level was set at 5%. All the analyses were performed using SPSS 20.0.

### Ethic statement

The research complied with current laws of Spain, Italy, and the European Union. Since the study was purely observational, we did not require the permission of the ethics committee of the University CEU Cardenal Herrera (Valencia, Spain).

## Results

We analysed 29 hours of video recordings and collected 433 yawning events (Y).

### Influence of age, sex, NDS and context on yawning frequency and duration

**Figure 2:**
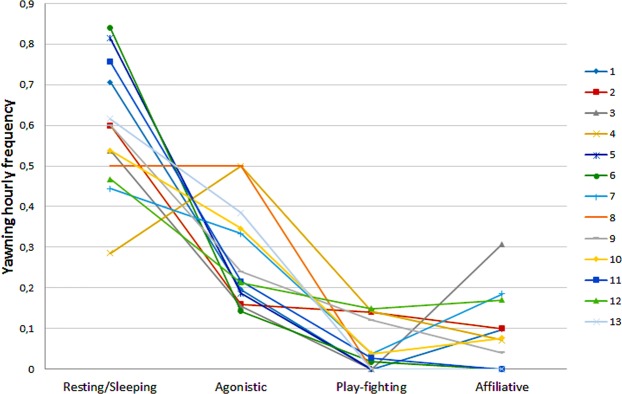
Distribution of the yawning hourly frequency for each subject of the colony.

Using LMM, we assessed which variables (fixed factors: age class; sex class, NDS values, context; random factor: animal identity) explained the distribution of the hourly frequency of yawning. We found a single best model which included only the variable “context” and explained 99% of the distribution (AICc = −56.70; F = 60.74, df1 = 3, df2 = 48, p = 0.0001) (Fig. 2). The AICc of the null model was 9.39 and the AICc of the full model was −40.82.

To verify which factors affected the mean yawn duration, we ran a LMM test. We found two competing models. The first model (AICc = 125.66) included the variables “context, age, context*age”, and had a 54% probability of being the best model. The second model (AICc = 126.29) included the variables “context, sex, context*sex”, and had a 40% of probability of being the best model. None of the variables had a significant effect. The third model (AICc = 130.82), including the variables “age, sex, age*sex”, cannot be discarded (ΔAICc < 6) (see Symonds and Moussalli^42^). The AICc of the null model was 140.80 and the AICc of the full model was 133.39.

### Yawning and self-scratching: Post-Conflict (PC) and Matched-Control (MC) observations

**Figure 3:**
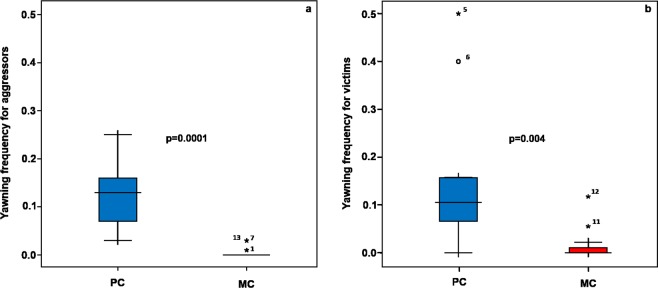
Yawning hourly frequency in the 30-sec time interval in Post-conflict (PC) and Matched-Control (MC) observations for aggressors (**a**) and victims (**b**). The box plots show the median and 25^th^ and 75^th^ percentiles; the whiskers indicate the values within 1.5 times the inter-quartile range, IQR. The open dot indicates an outlier more than 1.5 IQR from the rest of the scores. Asterisks indicate outliers more than 3.0 IQR from the rest of the scores.

**Figure 4:**
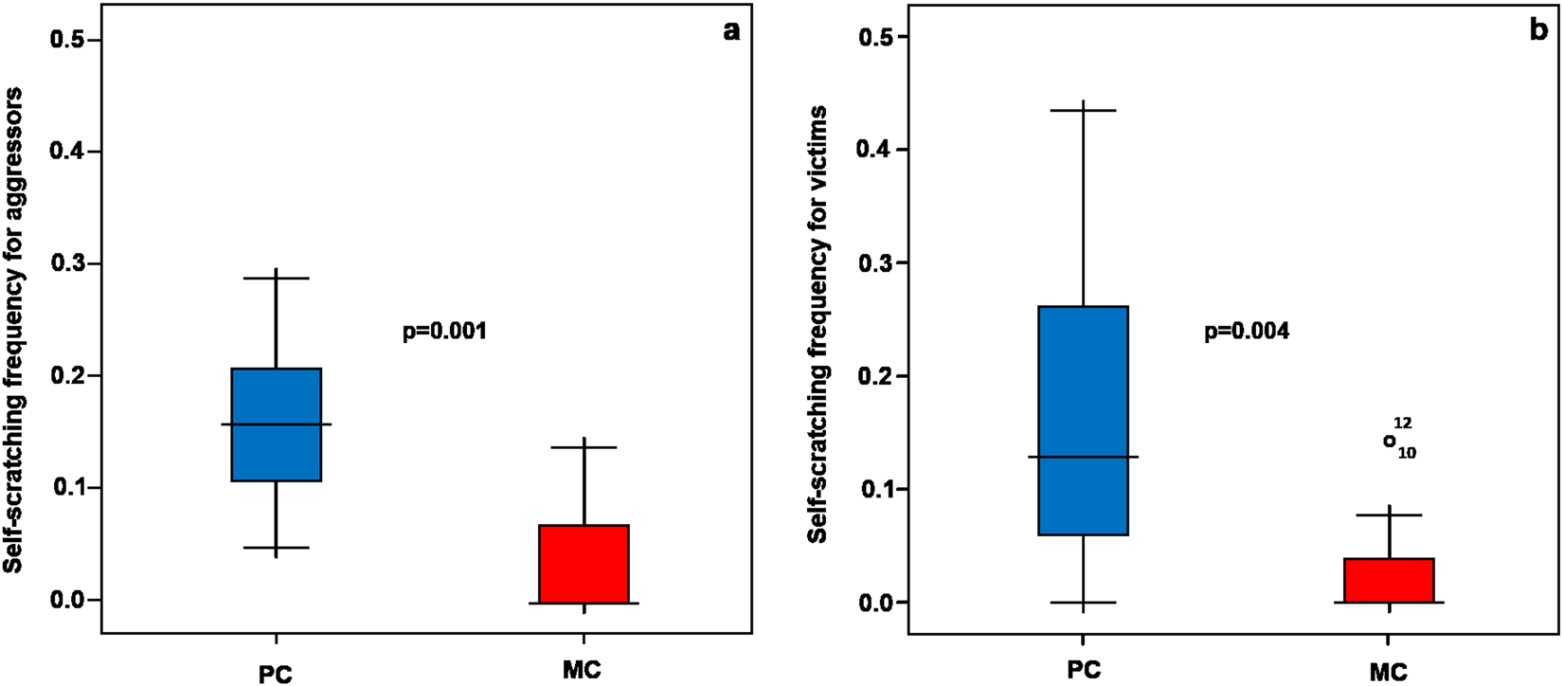
Self-scratching hourly frequency in the 30-sec time interval in Post-conflict (PC) and Matched-Control (MC) observations for aggressors (**a**) and victims (**b**). The box plots show the median and 25^th^ and 75^th^ percentiles; the whiskers indicate the values within 1.5 times the inter-quartile range, IQR. The open dot indicates an outlier more than 1.5 IQR from the rest of the scores.

We analysed the distribution of yawning for aggressors and victims within a 30-sec post-conflict time window (Fig. 3a,b). The Wilcoxon test revealed that the rates of Y performed in the post-conflict observation (PC) were significantly higher than the rates of yawns performed in the matched-control observation (MC) (T_aggressor_ = 0.00; N = 13; ties = 0; p = 0.0001; T_victim_ = 0.00; N = 12; ties = 3; p = 0.004) (Prediction 3 supported). When assessing the distribution of self-scratching (SCR) for aggressors and victims within the 30-sec post-conflict time window, SCR frequencies were significantly higher in the post-conflict (PC) than in matched-control (MC) for both the aggressor (T_aggressor_ = 3.00; N = 13; ties = 0; p = 0.001) and the victim (T_victim_ = 0.00; N = 12; ties = 3, p = 0.004) (Fig. 4a,b). Bonferroni correction (level of significance, p = 0.013). The analysis relied on a different number of subjects (aggressors = 13; victims = 12) because one of the subjects was never a victim of aggression.

## Discussion

The aim of this study was to test, for the first time, hypotheses on the potential functions of the spontaneous yawning in a marine mammal species, the South American sea lion (*Otaria flavescens*). We have to note that the hypotheses tested here are not necessarily mutually exclusive due to the multifunctional nature of yawning.

The LMMs revealed that the variable ‘context’ strongly affected the distribution of yawning frequency but did not have a strong effect on its mean duration. Yawning was mainly performed in the resting/sleeping context, when sea lions were lay on the platforms maintaining their eyes closed without engaging in any social behaviour with conspecifics (inactive state). They sometimes opened their eyes and, from time to time, moved their body parts. More rarely, their state of alertness was increased and they could interrupt the inactive phase by socially interacting with other sea lions nearby. Despite our difficulty in determining whether animals were actually sleeping, the sea lions often alternated long periods of total inactivity (eyes closed) with short periods of low activity (eyes opened). Our findings are consistent with the association of yawning with drowsiness already found in human and non-human primates^6,13–15,46,47^. These studies also reported that yawn distribution changed according to the sleep-wake rhythms of the different subjects with peaks of yawning during the transitional phases between the two states.

Sex does not seem to have a strong effect on either the frequency or duration of yawning, thus supporting the *Dimorphism Hypothesis*. In agreement with our results, in ring-tailed lemurs and Verreaux’s sifaka - two prosimian species showing monomorphism in canine and body size - yawning distribution did not significantly differ between sexes^13^. Although our results support the *Dimorphism Hypothesis* of yawning, due to the small number of males present in our study group, this support must be taken with caution. Due to the social structure of the species (one-male group or harem)^48^, the exploration of the *Dimorphism Hypothesis* of yawning in sea lions would need several data collections in different captive and wild groups to control for the possible male individual variability in yawning propensity and canine size.

Neither the frequency nor duration of yawning was influenced by the NDS values of the subjects (a quantitative measure of hierarchical dominance) thus confirming that in our species, dominants and subordinates do not show a strong bias in the levels of baseline anxiety as a function of a different inequity power distribution (*Resource Inequity Hypothesis* supported).To explain this finding the social features of South American sea lions have to be discussed. All the subjects of a colony strongly compete for shaded areas for thermoregulation, access to the sea for foraging, mating partners and for enough safe places for lactating/nursing^49^. Therefore, aggressive interactions are not limited to adults, as female aggression towards pups and infanticide attempts by sub-adult males have also been reported^38,49^. Therefore, the aggressive propensity characterizing the species^35^ can make each animal, independent of the role it takes in an agonistic encounter or its individual features (age, sex or rank), uncertain about the probability of being the recipient of aggression. This is in line with the findings on the distribution of self-scratching and yawning immediately after agonistic interactions. Self-scratching has been proven to be a reliable indicator of anxiety in human and non-human primates^26,50^, since it has been described in a wide range of stressful situations (*e*.*g*., crowded conditions, unfamiliar encounters, proximity of high-dominant individuals or post-conflicts)^51–54^. As for self-scratching, yawning has also been associated with the presence of environmental and social stressful stimuli^4,22,51,53^. In our study, we found that yawning and self-scratching significantly increased immediately after conflict for both the aggressor and the victim. This clearly indicates that both of these behavioural patterns are sensitive to social stressors and may have a similar function in restoring the homeostasis of the subjects independent of the role they play during the conflict.

In conclusion, our findings reveal that yawning is a multifunctional behaviour also seen in the South American sea lion. Spontaneous yawning seems to serve similar functions in different mammals living in well-structured social groups (*e*.*g*., primates, rodents and pinnipeds), independent of their phylogenetic distance and terrestrial/aquatic habits, thus suggesting that this behaviour may be a plesiomorphic trait.

## Supplementary information

Dataset 1

## Data Availability

All data generated or analysed during this study are included in this published article (and its Supplementary Information File).
